# Utilization of Seafood Processing By-Products for Production of Proteases by *Paenibacillus* sp. TKU052 and Their Application in Biopeptides’ Preparation

**DOI:** 10.3390/md18110574

**Published:** 2020-11-20

**Authors:** Chien Thang Doan, Thi Ngoc Tran, Van Bon Nguyen, Anh Dzung Nguyen, San-Lang Wang

**Affiliations:** 1 Department of Natural Science and Technology, Tay Nguyen University, Buon Ma Thuot 630000, Vietnam; dcthang@ttn.edu.vn (C.T.D.); ttngoc@ttn.edu.vn (T.N.T.); 2 Department of Chemistry, Tamkang University, New Taipei City 25137, Taiwan; 3 Institute of Biotechnology and Environment, Tay Nguyen University, Buon Ma Thuot 630000, Vietnam; nvbon@ttn.edu.vn (V.B.N.); nadzung@ttn.edu.vn (A.D.N.); 4 Life Science Development Center, Tamkang University, New Taipei City 25137, Taiwan

**Keywords:** angiotensin-I converting enzyme inhibitory activity, free radical scavenging activity, *Paenibacillus*, peptide, prebiotic, protease

## Abstract

Microbial fermentation of by-products is a renewable and efficient technique in the development of a range of useful products. In this study, protease synthesis by *Paenibacillus* sp. TKU052 was carried out on culture media containing some common seafood processing by-products (SPBPs) as the sole source of carbon and nitrogen (C/N). The most suitable C/N nutrition source for the production of proteases was found to be 3.0% (*w/v*) demineralized crab shells powder (deCSP) and maximal enzyme activity of 4.41 ± 0.16 U/mL was detected on the third day of the culture. Two proteases (P1 and P2) with a similar molecular weight of 31 kDa were successfully isolated and purified from the 3-day deCSP-containing medium. Both P1 and P2 exhibited the highest activity of gelatin hydrolysis at pH 6 and 60 °C. The gelatin hydrolysates catalyzed by *Paenibacillus* TKU052 proteases were evaluated for biological activities, including 2,2-diphenyl-1-picrylhydrazyl (DPPH) radical scavenging, angiotensin-I converting enzyme (ACE) inhibition, and prebiotic activities. The gelatin hydrolysates expressed 31.76–43.95% DPPH radical scavenging activity and 31.58–36.84% ACE inhibitory activity, which was higher than those from gelatin. Gelatin hydrolysates also showed the growth-enhancing effect on *Bifidobacterium bifidum* BCRC 14615 with an increase to 135.70–147.81%. In short, *Paenibacillus* sp. TKU052 could be a potential strain to utilize crab shell wastes to produce proteases for bio-active peptides’ preparation.

## 1. Introduction

Bioactive peptides are small fragments of proteins that have certain health benefits [1,2]. Until now, various bioactivities of these peptides, such as antioxidative, anti-cancer, angiotensin-I converting enzyme (ACE) inhibitory, anti-diabetes, and anti-microbial, have been explored [3,4,5,6,7,8]. Among the production methods of bioactive peptides, enzymatic hydrolysis is one of the most worth considering because of its advantages (easy to perform, short incubation time, and predictability). Proteases are major enzymes used for the preparation of biopeptides and various sources of this enzyme can provide different bioactive peptides [1,2].

Proteases are a group of enzymes capable of degrading protein directly to fragments of lower molecular weight (MW). These enzymes are produced by various living organisms, including microbes, plants, and animals [9,10]. Because of its widespread application, protease is one of the largest classes of industrial enzymes [11]. Among the protease-producing sources, microbes may provide several benefits, such as easy scalability for fermentation, less space requirement, and faster growth [10,11]. Besides, various protease-producing microbes can express the enzyme in high amounts in the medium using by-products as the nutrition source, for example, shrimp heads [9,10,11,12], squid pens [13,14], crab and shrimp shells [15], and agro-industrial wastes [16,17]. Among the by-product sources, seafood processing by-products (SPBPs) are considered a good candidate for producing protease because of the presence of protein in a significant proportion [9,10,11].

SPBPs comprise mainly fish wastes (skins, scales, viscera, frames, and heads), squid wastes (ink and pens), and shrimp and crab wastes (heads and shells) [6]. Among them, squid pens, shrimp shells, shrimp heads, and crab shells are chitinous wastes and are thereby widely used for the production of chitin [18,19,20,21,22,23]. However, the protein and mineral salts components in these materials are a significant impediment to the chitin production process. Hence, strong acids and alkalis are often used to remove those components, thereby releasing toxic water waste from the process. As a green technique, chitin-containing SPBPs could be utilized to produce several high-value products via microbial fermentation [24,25,26,27,28,29]. This gave rise to the idea of using these materials as cost-effective nutritional ingredients to produce protease by microbial fermentation in this study.

*Paenibacillus* was originally included in the genus of *Bacillus*, and later officially reclassified in 1993 as an individual genus [30]. Several important beneficial products of this genus have been discovered, including extra-cellular enzymes [9,10,31], antimicrobial substances [25,32], exopolysaccharides [25,33], anti-diabetic compounds [26,27], and antioxidants [25,28]. Interestingly, the production of proteases by *Paenibacillus* strains and their applications are rarely reported. Additionally, some *Paenibacillus* strains have shown good protease productivity on media using inexpensive by-products as nutritional ingredients [9,10,17]. This indicates the huge potential of producing proteases from *Paenibacillus* and the applications of this enzyme in various fields, e.g., biochemistry, food, and medicine.

In this study, *Paenibacillus* sp. TKU052, a protease-producing strain originally isolated from the soil of Tamkang University, was examined for its protease productivity on several SPBPs, such as demineralized crab shells powder (deCSP), squid pens powder (SPP), shrimp shell powder (SSP), demineralized shrimp shells powder (deSSP), and shrimp heads powder (SHP). Proteases were isolated and purified from the culture medium and then their properties were characterized. The protease-expression of *Paenibacillus* sp. TKU052 in different media was also analyzed by sodium dodecyl sulfate-polyacrylamide gel electrophoresis (SDS-PAGE). To determine the potential use, *Paenibacillus* sp. TKU052 proteases were used to catalyze gelatin to prepare bioactive peptides. Finally, gelatin hydrolysates were examined for 2,2-diphenyl-1-picrylhydrazyl (DPPH) radical scavenging activity, ACE inhibitory activity, and growth-enhancing effect on lactic acid bacteria.

## 2. Results and Discussion

### 2.1. Screening, Selection, and Identification of Protease-Producing Bacterium

SPP-containing medium was used to isolate protease-producing bacterial strains from soil samples collected from Tamkang University (New Taipei, Taiwan). Among the medium components, SPP containing a significant amount of protein and chitin is the only one that provides the carbon and nitrogen source. Thus, SPP-containing medium has been indicated as an efficient medium for screening protease- and chitinase-producing bacteria [10,12,18,19]. Among the isolated strains, TKU052 demonstrated the highest protease activity and was selected for further study. The morphological study showed TKU052 as a rod-shaped, gram-negative, and motile bacterium. The 16S rRNA sequence of TKU052 was found to be similar to *P. tyrfis* MSt1 (99.3%) and *P. elgii* SD17 (99.3%). However, the Analytical Profile Index (API) identification could not verify the species of TKU052. Therefore, the selected strain was simply called *Paenibacillus* sp. TKU052.

### 2.2. By-Products of Seafood Processing as the Sole C/N Source for Protease Production

**Figure 1:**
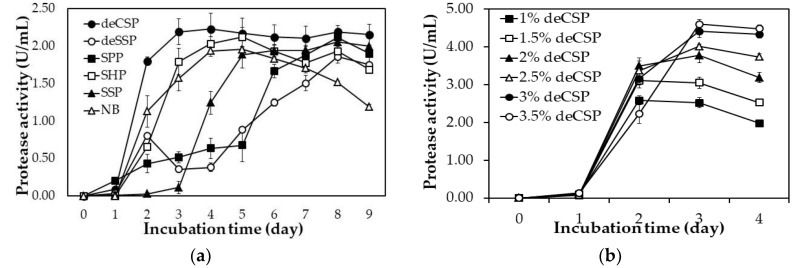
Effect of seafood processing by-products (SPBPs) (**a**) and concentration of demineralized crab shells powder (deCSP) (**b**) on the protease production of *Paenibacillus* sp. TKU052. The error bar is the standard deviation of three replications. SSP, shrimp shell powder; SPP, squid pens powder; SHP, shrimp heads powder; deSSP, demineralized shrimp shells powder; deCSP, demineralized crab shells powder; NB, nutrient broth.

In this study, several common types of SPBP were chosen for the investigation, including SHP, SPP, SSP, deSSP, and deCSP. One percent (*w/v*) of each type of SPBP was added to a basic mineral medium with MgSO_4_ (0.05%, *w/v*) and K_2_HPO_4_ (0.1%, *w/v*) to provide carbon and nitrogen nutrients for the action of *Paenibacillus* sp. TKU052. As shown in Figure 1a, the maximal protease activity of each medium was 2.23 ± 0.22 U/mL (deCSP, day 4), 1.86 ± 0.08 U/mL (deSSP, day 8), 2.12 ± 0.10 U/mL (SPP, day 8), 2.12 ± 0.13 U/mL (SHP, day 5), 2.06 ± 0.12 U/mL (SSP, day 8), and 1.96 ± 0.03 U/mL (NB, day 4). Moreover, the protease activity on days 2 and 3 of the deCSP-containing medium did not show any significant difference (2.19 ± 0.17 U/mL and 2.23 ± 0.22 U/mL, respectively). Thus, the deCSP-containing medium was found to be the most suitable for the protease synthesis by *Paenibacillus* sp. TKU052 base on the highest maximum enzyme activity with a shorter incubation time.

Several reports have defined the concentration of SPBP as a significant parameter for enzyme production [9,10]. Thus, the protease productivity of *Paenibacillus* sp. TKU052 was also determined on the medium containing deCSP at different concentrations (1%, 1.5%, 2%, 2.5%, 3%, and 3.5%). Figure 1b demonstrates the impact of deCSP on the productivity of protease by *Paenibacillus* sp. TKU052. A higher deCSP concentration was found to be more suitable for higher *Paenibacillus* sp. TKU052 protease productivity and the 3-day culture medium with 3% and 3.5% deCSP showed better protease activity compared with other deCSP concentrations with 4.41 ± 0.16 U/mL and 4.60 ± 0.13 U/mL of the enzyme activity, respectively (Figure 1b). In comparison, there was no significant difference between maximum protease activity at 3.0% or 3.5% deCSP, indicating that 3% deCSP could be an appropriate concentration for protease production at lower costs.

Crab shells are commonly known for the abundant release of chitinous by-products in the seafood processing industry [6]. Therefore, crab shells are considered as a raw material for the extraction of chitin [34]. Recently, because of the development of green techniques, crab shells have been used for the production of various bioactive products via microbial fermentation [26,35,36]. However, the application of this by-product as a cheap material for protease production using the *Paenibacillus* genus has rarely been reported. Hence, it was interesting to assess the conversion of deCSP to produce protease by *Paenibacillus* sp. TKU052.

### 2.3. Protease Purification

**Figure 2:**
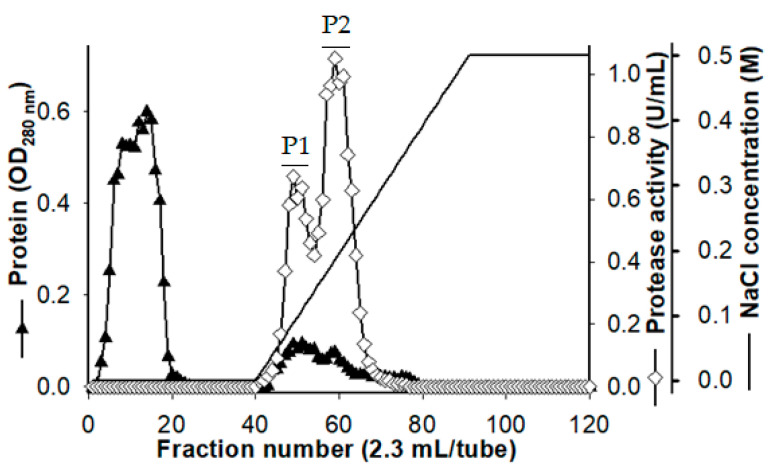
Ion-exchange chromatography profile of the *Paenibacillus* sp. TKU052 crude protease enzyme.

Two hundred and fifty milliliters of 3-day culture supernatant was used to purify the produced proteases under the following condition: Macro-Prep High S resin, 20 mM potassium phosphate (pH 6.0), and NaCl gradient from 0 M to 0.5 M. Two fractions with protease activity, named P1 and P2, were observed at the elution stage of the ion-exchange chromatography (Figure 2). Prior to the application of the next purification step, the P1 and P2 fractions were pooled, dialyzed against the buffer, and lyophilized. A high-performance liquid chromatography device with a KW-802 column was used for purification following the gel filtration method. Finally, the recovery yield of P1 and P2 fractions was 5.80% and 26.50% with 15.84 U/mg and 49.44 U/mg, respectively, of their specific activity (Table 1).

**Figure 3:**
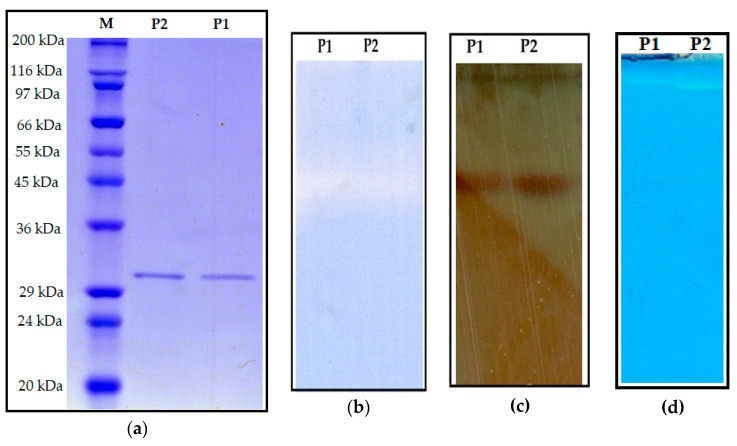
Sodium dodecyl sulfate-polyacrylamide gel electrophoresis (SDS-PAGE) (**a**), SDS-PAGE zymography (**b**), silver-stained zymography gel (**c**), and native-PAGE zymography (**d**) profiles of purified *Paenibacillus* sp. TKU052 protease.

The obtained proteases were examined for their homogeneity using SDS-PAGE and Coomassie blue staining. According to Figure 3a, both P1 and P2 showed a single band of 31 kDa (approximately). The obtained proteases also exhibited their in-gel proteolytic activity by zymogram analysis using gelatin as the substrate. One proteolytic band was observed at the same location in the lanes of both P1 and P2, on the gel (Figure 3b), indicating that two proteases have the same MW. Later on, the zymogram gel was silver-stained to re-confirm the homogeneity of the obtained proteases and only one protein band was observed at the location of the proteolytic band (Figure 3c). This indicates that the protease purification process was successful. *Paenibacillus* strains are often reported to secrete only one protease into the culture medium, however, some strains such as *P. polymyxa* SCE2 and *P. peoriae* NRRL BD-62 could produce four different proteases on thiamine, biotin, and nitrogen broth [9,10,14,37,38,39,40,41]. Notably, here, two proteases with the same MW (31 kDa) were isolated and purified from the culture supernatant of *Paenibacillus* sp. TKU052. Furthermore, the native-PAGE analysis revealed that P1 and P2 have different migration rates (Figure 3d). This result indicates that purified P1 and P2 are different isoenzymes. To the best of our knowledge, this could be the first report of protease isoenzymes from *Paenibacillus* strains. As such, the results of this study could be a valuable contribution to the existing knowledge about proteases from the genus *Paenibacillus.*

### 2.4. Effects of Temperature and pH on the Protease Activity and Stability

**Figure 4:**
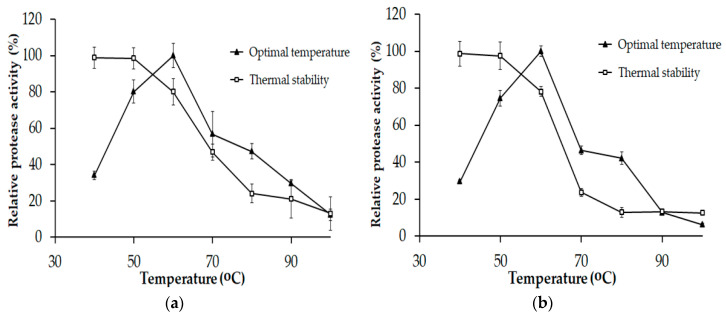
Optimal temperature and thermal stability of P1 (**a**) and P2 (**b**). The error bar is the standard deviation of three replications.

The optimal temperature of P1 and P2 was 60 °C and they were stable up to 50 °C (Figure 4). At the optimal temperature (60 °C), both P1 and P2 could retain nearly 80% of their original activities. Compared with other reports, *Paenibacillus* sp. TKU052 proteases exhibited a similar optimum temperature as that of protease from *P. mucilaginosus* TKU032 (60 °C) and higher than that of proteases from *P. tezpurensis* sp. nov. AS-S24-II and *P. lautus* [9,37,40]. However, its optimal temperature was lower than that of protease from *Paenibacillus* sp. TKU047 [10].

**Figure 5:**
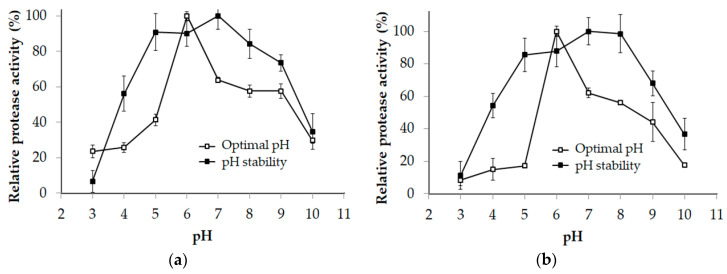
Optimal pH and pH stability of P1 (**a**) and P2 (**b**). The error bar is the standard deviation of three replications.

The influence of pH on the enzyme activity and stability of P1 and P2 was evaluated in the range of pH 3–10. Both P1 and P2 exhibited the highest activity at pH 6 and maintained over 80% of their activity in the range of pH 5–8 (Figure 5). This result differs from that reported in earlier studies, in which proteases from *Paenibacillus* strains exhibited optimal pH under alkaline conditions [9,10,14,37,38,39,40,41].

### 2.5. Effect of Metal Ion, Surfactants, and Protease Inhibitors

**Figure 6:**
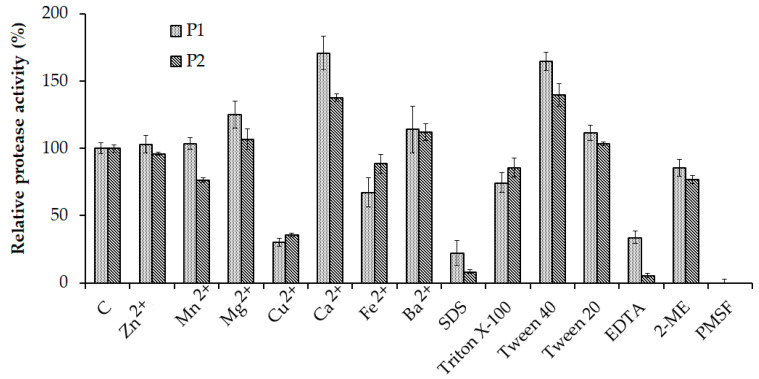
Effect of various chemicals on the activity of P1 and P2. The error bar is the standard deviation of three replications. EDTA, ethylenediaminetetraacetic acid; 2-ME, 2-mercaptoethanol; PMSF, phenylmethylsulfonyl fluoride.

Among the tested metal ions, the enzyme activity of P1 was enhanced by Mg^2+^ (125.11 ± 10.09%) and Ca^2+^ (170.78 ± 12.32%), while that of P2 was enhanced only by Ca^2+^ (137.83 ± 2.62%). Cu^2+^ showed a high inhibitory effect on the activity of both P1 (30.14 ± 2.96%) and P2 (35.49 ± 1.43%), whereas a moderate effect of Mn^2+^ on P2 (76.62 ± 1.57%) and Fe^2+^ on P1 (67.12 ± 10.79%) and P2 (88.63 ± 7.00%) was observed. SDS, a strong ionic surfactant, could dramatically decrease the enzyme activity of P1 to 22.37 ± 9.31% and P2 to 8.28 ± 1.38% relative to their normal activities. While Triton X-100 slightly reduced the activities of P1 (74.43 ± 7.30%) and P2 (85.75 ± 7.28%), tween 40 improved P1 and P2 activities by 164.84 ± 6.74% and 139.75 ± 8.21%, respectively. However, there was no significant difference in P1 and P2 activities by increasing tween 20, which were 111.41 ± 5.74% (P1) and 103.3 ± 1.40% (P2). Considering the protease inhibitors, phenylmethylsulfonyl fluoride (PMSF) and ethylenediaminetetraacetic acid (EDTA) showed high inhibitory effects on the activity of P1 and P2. They completely lost the activity in the presence of PMSF and their residual activity was only 33.79 ± 4.66% and 5.63 ± 1.57% (respectively) in the presence of EDTA. 2-mercaptoethanol (2-ME), a reducing agent, partially affected the enzyme activity by reducing it to 85.39 ± 6.36% (P1) and 76.83 ± 3.16% (P2) (Figure 6).

### 2.6. Substrate Specificity

**Figure 7:**
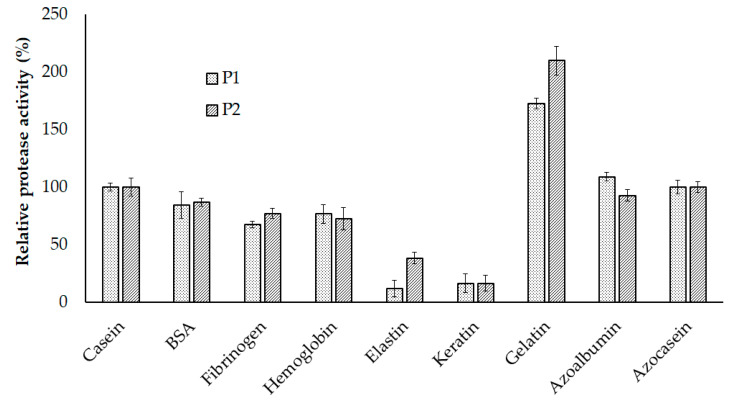
Substrate specificity of P1 and P2. The error bar is the standard deviation of three replications. BSA, bovine serum albumin.

The activity of the enzymes with casein as the control (100%) was used to examine enzyme specificity over other substrates, including bovine serum albumin (BSA), fibrinogen, hemoglobin, elastin, gelatin, and keratin. The result is displayed in Figure 7. Among these, P1 and P2 displayed the highest activity towards gelatin (172.58 ± 4.71% and 209.85 ± 12.43%, respectively) and the lowest towards elastin (12.32 ± 7.20% and 38.4 ± 5.04%, respectively), and keratin (16.49 ± 8.03% and 16.49 ± 6.82%, respectively). The activities of P1 and P2 on substrates including BSA (84.44 ± 11.41% and 87.00 ± 3.68%, respectively), fibrinogen (67.55 ± 3.08% and 77.08 ± 4.30%, respectively), and hemoglobin (76.82 ± 8.23% and 72.47 ± 8.23%, respectively) did not show any significant difference. Besides, no significant difference in the protease activity of P1 and P2 was observed towards azoalbumin (109.11 ± 3.90% and 92.72 ± 4.87%, respectively), and azocasein (100.00 ± 5.76% and 100.00 ± 4.46%, respectively).

### 2.7. Confirmation of Protease Production in Culture Medium

**Figure 8:**
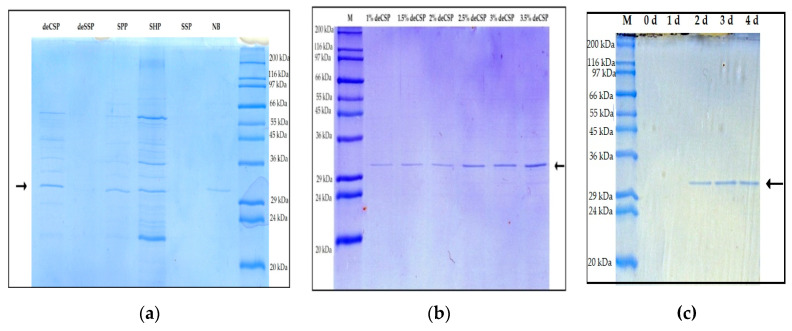
SDS-PAGE fingerprints of culture supernatants obtained from media containing different concentrations of SPBPs (**a**), deCSP (**b**), and from 3% deCSP with different incubation times (**c**): the position of protease bands.

Supernatants from various culture media of *Paenibacillus* sp. TKU052 were analyzed by SDS-PAGE and the band at 31 kDa was attributed to the position of the protease. As shown in Figure 8a, 31 kDa bands were clearly observed in the lanes of deCSP, SPP, SHP, and NB used as media for protease production. The intensities of the 31 kDa band in the lanes of deCSP and SHP were significantly higher than those of SPP and NB. The intensity of protease bands increased as deCSP concentrations increased (Figure 8b), indicating that the concentration of deCSP significantly affected protease production by *Paenibacillus* sp. TKU052. Further, the culture medium with 3% deCSP was analyzed. As shown in Figure 8c, no protease band was observed in the lanes with 0- and 1-day culture media, and the protease bands appeared from day 2, with maximum intensity on days 3 and 4. This is concurrent with the result of the protease activity presented in Figure 1. Thus, SDS-PAGE could be used to confirm the protease production of *Paenibacillus* sp. TKU052. Besides, the SDS-PAGE result also revealed that the intensity of the protease band was significantly higher than other proteins. This indicates that the protease is the predominant extracellular protein produced by *Paenibacillus* sp. TKU052 on deCSP-containing medium. It is essential to improve protease production to meet the current biotechnological advancements. deCSP, a cheap material from seafood processing by-product, could be considered as a potential process for larger-scale application using *Paenibacillus* sp. TKU052 because it produces protease efficiently.

### 2.8. Gelatin Hydrolysis

**Figure 9:**
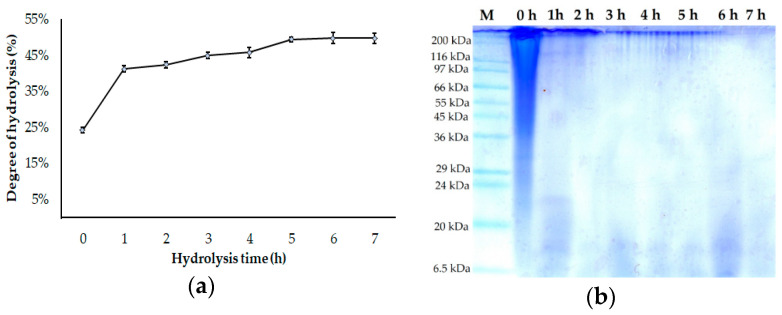
Degree of hydrolysis (**a**) and SDS-PAGE profile (**b**) of gelatin hydrolyzed by *Paenibacillus* sp. TKU052 proteases. The error bar is the standard deviation of three replications.

Gelatin was observed to be the most suitable substrate for the *Paenibacillus* sp. proteases; therefore, its hydrolysis was conducted and analyzed as the degree of hydrolysis (DH) and by SDS-PAGE. The proteases obtained from the ion-exchange chromatography step were used to catalyze gelatin hydrolysis. As shown in Figure 9a, the rate of gelatin hydrolysis was high within the first hour (from 24 ± 0.77% at 0 h to 41.22 ± 0.96% at 1 h). The rate of enzymatic hydrolysis was progressively diminished from 1 h to 5 h (41.22 ± 0.96% to 49.36 ± 0.65%) until reaching a stationary stage when no obvious hydrolysis occurred from 5 h to 7 h (49.36 ± 0.65% to 49.69 ± 1.50%). A similar hydrolysis pattern was observed in earlier reports [42]. Gelatin hydrolysis by *Paenibacillus* sp. TKU052 proteases was also confirmed by SDS-PAGE (Figure 9b). Before the hydrolysis took place (represented as 0 h), gelatin appeared as a broad area over the examined MW (6.5 kDa–200 kDa), in which the area with the highest intensity had MW > 36 kDa. After 1 h of hydrolysis, a broad area with MW < 30 kDa appeared with a greatly reduced intensity of the gelatin polypeptides. The result indicated that the rate of hydrolysis was high in the first hour to release the lower MW fragments. This is consistent with the DH results described above. From 2 h onwards, the highest intensity areas were observed at MW < 20 kDa, indicating the appearance and release of peptides at a high concentration from gelatin hydrolysis. The presence of peptides as the major component in gelatin hydrolysate may potentially result in several biological activities such as anti-oxidative, ACE inhibitory, or prebiotic activities. Therefore, the gelatin hydrolysates catalyzed by the mixture of *Paenibacillus* sp. TKU052 proteases were further investigated for their biological activity, as discussed in the following section.

### 2.9. Bioactivity Evaluation of Gelatin Hydrolysate

**Figure 10:**
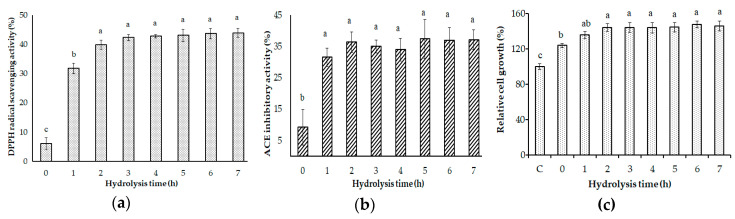
Evaluation of the 2,2-diphenyl-1-picrylhydrazyl (DPPH) radical scavenging activity (**a**), angiotensin-I converting enzyme (ACE) inhibitory activity (**b**), and the growth-enhancing effect on *B. bifidum* BCRC 14615 (**c**) of gelatin hydrolysates. The error bar is the standard deviation of three replications. The letters a, b, and c indicate significant differences based on Tukey’s HSD (honestly significant difference) test with *p* < 0.05.

In aerobic metabolism, oxidation is a significant process; however, it may lead to free radical formation. Nucleic acids, lipids, and proteins could be damaged, leading to cell death and disruption to tissues by the harmful impact of free radicals. Thus, antioxidant-containing foods could be used to support and defend the human body against free radicals [22,43]. In the search for a potential antioxidant, peptides are a well-known source and are gradually being widely recognized. In this study, the antioxidant activity of gelatin hydrolysates was examined using the DPPH radical scavenging method. The result revealed that gelatin by itself showed nearly no DPPH radical scavenging activity (6.00 ± 2.14%), while all gelatin hydrolysates exhibited this ability. Gelatin hydrolysates had high activity in the first 2 h of incubation (31.76 ± 1.81% at 1 h and 39.80 ± 1.60% at 2 h); however, further incubation (from 2 h to 7 h) did not increase the activity (39.80 ± 1.60%, 42.38 ± 1.04%, 42.84 ± 0.57%. 43.12 ± 2.10%, 43.67 ± 1.76%, and 43.95 ± 1.59%, respectively) (Figure 10a). The result suggests that *Paenibacillus* sp. TKU052 proteases degraded gelatin to produce the proton-effective peptides that possibly reacted with unstable free radicals in DPPH to turn them into more stable products and terminate the radical chain reaction.

The angiotensin-I converting enzyme (ACE) plays a critical role in the control of blood pressure via the renin-angiotensin and kinin-kallikrein processes. Thus, ACE inhibition may lead to a decrease in blood pressure and thereby prevent hypertension [44,45]. In this study, gelatin hydrolysates were also examined the ACE inhibitory activity and the results are displayed in Figure 10b. The ACE inhibitory activity of gelatin was 9.21 ± 5.62% and it increased dramatically after the hydrolysis occurred. There was no significant difference in the ACE inhibitory activity of all hydrolysates from 1 h to 7 h (31.58 ± 2.91%, 36.32 ± 3.24%, 35.00 ± 2.07%, 33.95 ± 3.55%, 37.37 ± 6.19%, 36.84 ± 4.23%, and 37.11 ± 3.18%, respectively). This indicates that the incubation for the hydrolysis of gelatin to obtain ACE inhibitory peptides could be performed for a short time (1 h) using the mixture of *Paenibacillus* sp. proteases to catalyze hydrolysis.

Prebiotics are food compounds that allow beneficial microorganisms such as bacteria and fungi to develop or function [46]. The results of prebiotics on *Bifidobacteria* and *Lactobacillus* have been the key subject of their studies [47]. In this study, the growth-enhancing effect of gelatin hydrolysates on lactic acid bacteria was examined on five lactic acid bacterial strains, including *B. bifidum* BCRC 14615, *L. rhamnosus* BCRC 16000, *L. rhamnosus* BCRC 10940, *L. paracasei* subsp. *paracasei* BCRC 14023, and *L. lactis* subsp. *lactis* BCRC 10791. Gelatin hydrolysates did not show a significant effect on the growth of *L. rhamnosus* BCRC 16000, *L. rhamnosus* BCRC 10940, *L. paracasei* subsp. *paracasei* BCRC 14023, and *L. lactis* subsp. *lactis* BCRC 10791 (data not shown). Interestingly, gelatin and gelatin hydrolysates exhibited a clear effect on the growth of *B. bifidum* BCRC 14615. The result is presented in Figure 10c, showing an increase of 123.90 ± 2.57% of the bacterial growth in the gelatin-containing medium. Notably, gelatin hydrolysates showed a higher growth enhancement effect on *B. bifidum* BCRC 14615 than gelatin. Particularly, increases to 135.70 ± 4.08%, 144.36 ± 4.44%, 144.26 ± 5.43%, 143.84 ± 6.06%, 144.57 ± 5.37%, 147.81 ± 3.84%, and 145.93 ± 5.54% of the bacterial growth were observed in the medium at 1 h, 2 h, 3 h, 4 h, 5 h, 6 h, and 7 h gelatin hydrolysates, respectively. This indicates that the gelatin hydrolysates catalyzed by *Paenibacillus* sp. TKU052 protease may be potentially used as a prebiotic. Various types of proteins, hydrolysates, and peptides have demonstrated growth-enhancing effects on probiotics [48]; however, gelatin and gelatin hydrolysates have received little attention until now. Thus, this result could be a valuable contribution to the investigation of the potential of gelatin hydrolysates as a prebiotic source.

## 3. Materials and Methods

### 3.1. Materials

Azocasein, DPPH, lung acetone powder from rabbit, 2-ME, tyrosine, phenylmethylsulfonyl fluoride, and *N*-Hippuryl-His-Leu (HHL) were bought from Sigma Co. (St. Louis, MO, USA). DeMan, Rogosa, and Sharpe (MRS) broth as well as nutrient broth (NB) medium were bought from Himedia (Mumbai, India). Lactic acid bacterial strains (including *B. bifidum* BCRC 14615, *Lactobacillus rhamnosus* BCRC 16000, *L. rhamnosus* BCRC 10940, *L. paracasei* subsp. *paracasei* BCRC 14023, and *Lactococcus lactis* subsp. *lactis* BCRC 10791) were obtained from Bioresource Collection and Research Center (Hsinchu, Taiwan). Shrimp heads were procured from Fwu-Sow Industry (Taichung, Taiwan), whereas other SPBPs (crab shells, squid heads, and shrimp shells) were from Shin-Ma Frozen Food Co. (I-Lan, Taiwan). The demineralization of shrimp shells and crab shells was carried out as described earlier [9]. The other chemicals used were of the highest possible quality.

### 3.2. Screening, Selection, and Identification of the Protease-Producing Bacterium

SPP medium containing 1% (*w/v*) SPP, 0.05% (*w/v*) MgSO_4_, and 0.1% (*w/v*) K_2_HPO_4_ was used to isolate the protease-producing bacteria [10]. Soil samples collected from Tamkang University (New Taipei, Taiwan) were diluted by sterile 0.9% (*w/v*) saline solution and spread on the SPP-containing medium agar dish. After two days of incubation at 37 °C, single colonies were obtained, which were then incubated in 250 mL flasks with 100 mL of SPP-containing medium for three days at 37 °C and 150 rpm shaking speed. The culture medium was collected for the estimation of protease activity. The strain showing the highest protease productivity was selected for characterization, and identification of its scientific name by morphological, biochemical, and 16S rDNA sequences analysis.

### 3.3. Protease Assay

A sample volume of 50 µL was added into a microfuge tube (Eppendorf) containing 50 µL azocasein (1%, *w/v*) and the mixture was immediately incubated at 37 °C for 30 min. Then, 300 µL trichloroacetic acid solution (5%, *w/v*) was added to the mixture to stop the reaction and precipitate the residual azocasein. This precipitate was separated from the supernatant by centrifugation (13,000 rpm, 20 min). Finally, 300 µL of the supernatant was transferred to a 96-well plate and its absorbance was measured at 420 nm using an enzyme-linked immunosorbent assay (ELISA) plate reader. One protease unit was defined as an increase in A_420 nm_ by 0.01 in 1 min under the assay condition.

### 3.4. By-Products of Seafood Processing as the Sole C/N Source for Protease Production

To prepare a medium containing SPBP, 1% of each SPBP was added into 100 mL of basal medium containing MgSO_4_ (0.05%, *w/v*) and K_2_HPO_4_ (0.1%, *w/v*). The fermentation of *Paenibacillus* sp. TKU052 on different SPBP-containing medium was initiated by adding 1 mL of bacterial seed solution to each medium and incubated at 37 °C and 150 rpm shaking. The protease activity of the culture medium was checked every 24 h during the incubation time. To investigate the optimal deCSP concentration for the protease production of *Paenibacillus* sp. TKU052, a range of deCSP amounts (1 g, 1.5g, 2 g, 2.5 g, 3 g, and 3.5 g) were added to 100 mL of basal medium. The conditions for the fermentation were described above.

### 3.5. Enzyme Purification

The culture medium was centrifuged at 6000 rpm for 30 min to remove the residual solids and bacterial cells. The liquid was then mixed with ammonium sulfate (60% *w/v*) and kept at 4 °C for 24 h. The precipitate that appeared in the mixture of culture supernatant and ammonium sulfate was conveniently obtained by centrifugation (9000 rpm, 30 min). A small volume of 20 mM potassium phosphate buffer (PPB) at pH 6 was used to dissolve the obtained precipitate. The residual ammonium sulfate was removed by dialyzing the solution against PPB for 24 h using a cellulose membrane (cellusep T2, Interchim, Montluçon, France). For the enzyme purification, the crude enzyme was loaded onto the Macro Prep High S column (Biorad, Hercules, CA, USA) pre-equilibrated with PPB (20 mM, pH 6). The washing step took place until A_280 nm_ reached a stable value. After the washing step, a linear gradient of NaCl (0 M–0.5 M) was conducted to elute the target enzymes. Fractions exhibiting protease activity were then pooled, and concentrated by the freezing-drying method. Finally, the enzyme was purified by size exclusion chromatography (KW-802.5 column, Showa Denko K.K., Tokyo, Japan). The protein content was estimated by the Lowry method [37].

### 3.6. Sodium Dodecyl Sulfate-Polyacrylamide Gel Electrophoresis (SDS-PAGE) Analysis

SDS-PAGE was conducted according to the method of Laemmli [49] on a 10% resolving gel. The sample was prepared in the sample buffer containing 2-ME and SDS, and the mixture was heated at 100 °C for 2 min. Then, 10 µL sample/well was loaded for electrophoresis at 25 mA, after which the gel was stained by Protein Assay Dye Reagent (Bio-Rad, Berkeley, CA, USA) and then de-stained by methanol/acetic acid/water solution (1/1/8, *v/v/v*). The visual band of the enzyme was compared with the bands of protein markers to determine its MW.

### 3.7. Zymography

The resolving gel was co-polymerized with 0.02% gelatin (*w/v*). The enzyme sample was prepared in the sample buffer containing SDS and 10 µL of the sample was loaded per well for electrophoresis at 114 V and 4 °C. The gel was then washed by 2.5% Triton X-100 (prepared in PPB), followed by PPB alone. The proteolytic reaction was carried out by incubating the gel in PPB for 12 h at 37 °C. The staining and de-staining steps were conducted following the SDS-PAGE method described above. The protease activity band appeared as a clear band against the blue background of the gel.

### 3.8. Effect of Temperature and pH

To determine the optimal temperature for protease activity, the reaction was conducted at different temperatures (40–100 °C). The thermal stability of the enzyme was determined based on the residual activity after pre-treating it at different temperatures for 1 h. To determine the optimal pH, the protease assay was conducted at different pH (pH 3–10) by adjusting the pH of the reaction solution. The pH stability of the enzyme was determined based on its residual activity after pre-treating it at different pH for 1 h. The buffer system used to adjust the pH of the enzyme solution included sodium carbonate buffer (pH 9–10), PPB (pH 6–8), sodium acetate buffer (pH 5), and glycine HCl buffer (pH 3–4).

### 3.9. Substrate Specificity

The protease substrates that were used included native proteins such as casein, elastin, myoglobin, fibrinogen, hemoglobin, bovine serum albumin (BSA), keratin, and gelatin, as well as synthesis proteins (azocasein and azoalbumin). Casein was used as the control to test the substrate specificity of P1 and P2 against native proteins, whereas azocasein was used as the control in the case of synthesis proteins. Protease assay using casein was carried out as described earlier [9].

### 3.10. Effect of Metal Ions, Inhibitors, and Surfactants

Metal ions, 2-ME, PMSF, and EDTA were prepared at a concentration of 5 mM, while SDS, tween 20, and tween 40 were prepared at 10%. Each chemical solution was added to the enzyme solution and the mixture was kept for 30 min. Later on, the substrate was added into the mixture to determine the residual enzyme activity. 

### 3.11. Gelatin Hydrolysis

The enzyme solution used in this experiment was a mixture of P1 and P2 fractions obtained from the ion-exchange chromatography step. Gelatin was prepared at a concentration of 1% (*w/v*) in PPB (pH 6) and the hydrolysis was performed for 0–7 h at 60 °C. At the end of hydrolysis, the reaction solution was heated at 100 °C for 30 min to eliminate any enzyme activity. The gelatin hydrolysate was then dried by the freezing-drying method. The DH of gelatin hydrolysate was detected based on the quantification of the soluble protein [50]. The SDS-PAGE analysis of gelatin hydrolysate was according to the SDS-PAGE protocol described above.

### 3.12. Evaluation of Gelatin Hydrolysate Bioactivity

The gelatin hydrolysate in this study was tested for its DPPH radical scavenging activity, ACE inhibitory activity, and growth-enhancing effect on lactic acid bacteria.

DPPH radical scavenging activity assay was carried out according to the previous report [51]. In short, a sample volume of 50 µL was added into a microfuge tube containing 250 µL DPPH solution (1 mM in methanol), and the mixture was immediately kept in the dark for 20 min. To prepare the control, a 50 µL sample was replaced by 50 µL distilled water. Then, the violet color of the mixture was measured at 517 nm using an ELISA plate reader. The DPPH radical scavenging activity of the sample was determined by the following formula:

DPPH radical scavenging activity = 100 × (A_C_ − A_S_)/A_C_ (%) (1)

where A_C_ is the absorbance at 517 nm of the control and A_S_ is the absorbance at 517 nm of the sample.

ACE inhibitory activity assay was according to the method of Cushman and Cheung (1971) [52]. In short, a sample volume of 20 µL was added into a glass tube containing 50 µL of 8 mM HHL, and then 10 µL of the ACE solution was added to that mixture. The tube was gently shaken and mounted on an incubator (37 °C, 60 min). The ACE activity was eliminated by adding 62.5 µL HCl (1 M). To extract the product of the reaction (hippuric acid), an amount of ethyl acetate (375 µL) was added to the tube and then the mixture was vortexed for 15 s. The ethyl acetate layer was collected, dried, and re-dissolved by 4 mL of distilled water. Later on, a UV/vis spectrophotometer was used to measure the absorbance of the hippuric acid solution (228 nm). To prepare the control, the sample (20 µL) was replaced by 20 µL distilled water. To prepare the blank, the HCl solution was added to the mixture of the ACE solution, sample, and substrate before the ACE activity took place. The ACE inhibitory activity was determined by the following formula:

ACE inhibitory activity = 100 × [(A_C_ − A_CB_) − (A_S_ − A_SB_)/ (A_C_ − A_CB_)] (%) (2)

where A_C_ is the absorbance at 228 nm of the control, A_S_ is the absorbance at 228 nm of the sample, A_CB_ is the absorbance of the control blank, and A_SB_ is the absorbance at 228 nm of the sample blank.

In the DPPH radical scavenging and ACE inhibitory assays, the sample concentration was prepared at 10 mg/mL.

The growth-enhancing effect of gelatin hydrolysate was conducted on five lactic acid bacteria strains (*B. bifidum* BCRC 14615, *L. rhamnosus* BCRC 16000, *L. rhamnosus* BCRC 10940, *L. paracasei* subsp. *paracasei* BCRC 14023, and *L. lactis* subsp. *lactis* BCRC 10791). MRS was used as the basal medium and the gelatin hydrolysate 0.1% (*w/v*) concentration was added to the MRS medium. The bacterial cultivation was maintained at 37 °C for 24 h and the bacterial growth was measured at A_600 nm_ using an ELISA reader. The A_600 nm_ of the culture MRS medium was used as the control to calculate the relative cell growth of the bacterium on culture MRS containing gelatin hydrolysate. In the case of *B. bifidum* BCRC 14615, the medium was supplemented with 0.05% cysteine, and the cultivation was carried out in the anaerobic condition.

## 4. Conclusions

The study on the development of seafood processing by-products into high-quality products is ongoing. A low-cost medium containing deCSP as the sole carbon and nitrogen nutrient was found to be highly effective in the production of *Paenibacillus* sp. TKU052 proteases. From the deCSP medium, two proteases with a similar MW of 31 kDa were isolated, purified, and characterized. The hydrolysis of gelatin catalyzed by *Paenibacillus* sp. TKU052 proteases exhibited DPPH radical scavenging, ACE inhibitory, and prebiotic activities. Therefore, the proteases obtained from the conversion of deCSP by *Paenibacillus* sp. TKU052 may be potentially useful in biotechnology, food, and medicinal applications.

## Figures and Tables

**Table 1:** A summary of the purification of the proteases from *Paenibacillus* sp. TKU052.

Step	Total Protein (mg)	Total Activity (U)	Specific Activity (U/mg)	Recovery (%)	Purification (fold)
**Cultural supernatant**	1557.35	162.5	0.10	100.00	1.00
**(NH4)_2_SO_4_ precipitation**	174.21	148.2	0.85	91.20	8.15
**Ion-exchange chromatography**					
**P1**	2.16	24.91	11.52	15.33	110.41
**P2**	1.83	56.23	30.74	34.60	294.61
**Gel filtration**					
**P1**	0.59	9.42	15.84	5.80	151.83
**P2**	0.87	43.07	49.44	26.50	473.82
